# Influence of motivational interviewing on postoperative mobilization in the enhanced recovery after surgery (ERAS®) pathway in elective colorectal surgery - a randomized patient-blinded pilot study

**DOI:** 10.1007/s00423-024-03321-z

**Published:** 2024-04-22

**Authors:** Rico Wiesenberger, Julian Müller, Mario Kaufmann, Christel Weiß, David Ghezel-Ahmadi, Julia Hardt, Christoph Reissfelder, Florian Herrle

**Affiliations:** 1 Department of Surgery, Universitätsmedizin Mannheim, Medical Faculty Mannheim, Heidelberg University, Theodor-Kutzer-Ufer 1-3, 68167 Mannheim, Germany; 2 Institute for Medical Statistics, Universitätsmedizin Mannheim, Medical Faculty Mannheim, Heidelberg University, Theodor-Kutzer-Ufer 1-3, 68167 Mannheim, Germany; 3 Department of Anaesthesiology and Critical Care Medicine, Universitätsmedizin Mannheim, Medical Faculty Mannheim, Heidelberg University, Theodor-Kutzer-Ufer 1-3, 68167 Mannheim, Germany; 4 DKFZ-Hector Cancer Institute at the University Medical Center Mannheim, Mannheim, Germany

**Keywords:** Minimal-invasive surgery, daVinci, Movisens, Colorectal cancer, Motion sensors

## Abstract

**Purpose:**

Early mobilization is an essential component of the Enhanced Recovery after Surgery (ERAS®)-pathway. However, a large percentage of patients fail to achieve the ERAS**®** recommended goal (360 min out of bed from post-operative day 1/POD1). Motivational Interviewing (MI) is an evidence-based type of patient-centered consultation to promote intrinsic motivation. This study aims to evaluate if MI can improve postoperative mobilization.

**Methods:**

This two-arm, patient-blinded pilot randomized controlled trial included ERAS**®**-patients undergoing elective bowel resections. Conversations were validated by MI Treatment Integrity. Two validated motion sensors (movisens) and self-assessments were used to measure mobilization (POD1–POD3: Time out of bed, time on feet and step count).

**Results:**

97 patients were screened, 60 finally included and randomized. Cumulatively across POD1–3, the intervention group (IG) was longer out of bed than the control group (CG) (median: 685 vs. 420 min; *p*=0.022). The IG achieved the ERAS**®**-goal of 360 min/day more frequently across POD1–3 (27.4% vs. 10.61%; *p*=0.013). Time on feet was 131.5 min/day (median per POD) in IG vs. 95.8 min/day in the CG (*p*=0.212), step count was 1347 in IG vs. 754 steps/day in CG (*p*=0.298).

**Conclusion:**

MI could be conducted low threshold and was well accepted by patients. MI can improve mobilization in the context of ERAS**®**. Despite better performance, it should be noted that only 27.4% of the IG reached the ERAS**®**-compliance goal of 360 min/day. The findings of this pilot study stipulate to further test the promising perioperative effects of MI within a multicenter superiority trial.

**Registration:**

This study was registered prospectively in the German Clinical Trials Register on 25.02.2022. Trial registration number is “DRKS00027863”.

**Supplementary Information:**

The online version contains supplementary material available at 10.1007/s00423-024-03321-z.

## Introduction

Perioperative management through Enhanced Recovery after Surgery (ERAS**®**) includes multiprofessional cooperation according to evidence-based treatment pathways [1, 2]. ERAS**®** rapidly restores physiological homeostasis after surgery and accelerates the entire recovery of patients [3]. Successful implementation of ERAS**®** has a reduced overall complication rate of up to half and a median two to three days shorter duration of inpatient stay, compared to classic perioperative management in bowel surgery [3–5]. Early mobilization (Σ 360 min out of bed from post-op day 1 / POD1) is an essential success factor of the ERAS**®**-protocol for colorectal surgery [6, 7]. It has been shown that more postoperative mobilization is associated with fewer complications and shorter hospitalization [8]. Since postoperative activity is largely determined by patient compliance and motivation, achieving the mobilization-goal in ERAS**®**-clinics is a major challenge [4, 8, 9]. Studies have shown that more than half of ERAS**®**-patients don’t reach the daily goal of 360 min out of bed [10, 11]. Nevertheless, there is little research so far on how postoperative mobilization can be improved.

Motivational Interviewing (MI) [12] is an evidence-based, patient-centered conversation style that combines various psychological approaches. The most common core competencies include among other things open-ended questions, active listening, summarizing and appreciation [13]. The application of the MI-principles enhances intrinsic motivation as well as compliance of patients [14]. The MI basic skills can be learned in a few days [15, 16], so that MI seems suitable as a low-threshold intervention. A few reviews have shown effectiveness of MI in various medical fields. MI is already established as an effective method for treating substance abuse and addictions [17], but there are also positive effects in areas such as dental care, HIV viral load, systolic blood pressure and body weight [18]. In addition, several studies have found that MI can increase physical activity in a wide variety of study settings [18–21]. Currently there are only isolated studies concerning the use of MI in surgery. Referring to this, MI showed positive effects on diet motivation after bariatric surgery [22], reduced opiate use [23] and general rehabilitation after orthopedic surgery [24]. New and not investigated yet is the use of MI to support the recovery process after bowel surgery.

The aim of the present study was to examine the integration of MI into the ERAS**®**-concept and whether it can improve postoperative mobilization.

## Material & methods

### Participants

Patients undergoing elective bowel surgery at the University Hospital Mannheim were eligible.

The inclusion criteria were ≥18 years, cognitive abilities for informed consent, Barthel index ≥10 points for mobility (assisted walking >50 meters). Reasons for exclusion were inability to have a fluent conversation (language barrier, hearing problems, mental states), previous study participation, PEG or parenteral nutrition, cardiac devices and expected incompliance concerning the protocol requirements (especially handling with technical devices).

A sample size of 50 patients was planned for this pilot-study.

### Study design & procedure

This study is part of the MINT-ERAS**®**-project, a single-center, randomized, pilot-trial including two arms with patient-blinding.

Participants were randomized equally into intervention (IG) and control group (CG). The computer-generated randomization list in blocks of four (2 intervention and 2 control) was created by the SAS procedure "PROC PLAN". Randomization of the participants occurred on their date of premedication, which was scheduled by non-involved staff after study inclusion. Both study groups were treated by the same standards within the certified ERAS**®**-pathway for colorectal surgery at the Universitätsmedizin Mannheim [25]. The patients received an informative ERAS**®**-consultation preoperatively, which is conducted by the ERAS**®**-nurse who is specifically responsible for the ERAS**®**-patients and visits them daily after surgery (excluding weekends). The ERAS**®**-nurse was aware of which patients are participating in the study; however, she didn’t know the group allocation.

In the IG, MI techniques were used in pre- and postoperative visits, whereas MI was not used in visits of the CG. During the CG visits, mainly closed questions were asked (e.g. "Are you fine?"; "Have you been active today?") and active reflection was avoided. An example of a MI conversation is given in appendix 1. Both study groups were supervised by the same two persons (RW and JM) and visited in the same frequency (see Table 1). The same topics were addressed during the visits in both groups. Preoperatively, the focus was on sensor handling and ERAS**®**-goals; postoperatively, the focus was on daily activities, checking sensors and general data collection (pain, activity, etc.). POD0–3 visits were once daily between 6:00 pm and 8:30 pm. On POD0, the study participants were provided with the motion sensors, which were worn throughout the study period and removed on POD3 after 8 pm. Follow-up via phone was done 28–32 days after surgery to complete questionnaires.

**Table 1:** Study visits on top to the regular ERAS®-nurse consultations

Visit	1	2	3	4	5	6
Day	A few days before surgery	Day 0 (Surgery)	post-operative day 1	post-operative day 2	post-operative day 3	post-operative day 28-32
~time expenditure [min]	20	15				10
location	Outpatient clinic	hospital				phone

### Motivational Interviewing validity

The MI interviews were conducted by two persons (RW and JM) who completed a three-day certified basic course in MI [26]. Prior to the recruitment start, a learning curve evaluation was performed under study conditions in which each of the MI-interviewers cared for a patient using MI. The MI consultations of this training phase were video-recorded and served as a basis for supervision by experienced MI coaches (GK Quest Heidelberg) [27]. After data collection, four audio recordings from each of the two interviewers (3x intervention pre-op/day0/day1; 1x control) were analyzed by a modified version of the Motivational Interviewing Treatment Integrity (MITI) [28] and Client Language Easy Rating (CLEAR) [29]. All 8 recordings are conversations with different patients. 15 minutes of each consultation were evaluated. Since the 15-minute length wasn’t reached for one POD1 interview, the missing length was added with the beginning of the POD2 interview. Additionally, for the first three MI-visits (pre-op, POD0, and POD1), a guideline containing questions and MI-elements was developed and subtly followed by the interviewers (see appendix 2). However, because MI can only be standardized to a certain extent, there were large individual differences in how many and which elements of this guideline were used during each interview. The percentage use of the elements was surveyed (see appendix 2).

### Motion sensors

For objective physical activity and body position tracking, motion sensors recorded data from 23:59 pm on POD0 for 68 hours until 8:00 pm on POD3.

**Fig. 1:**
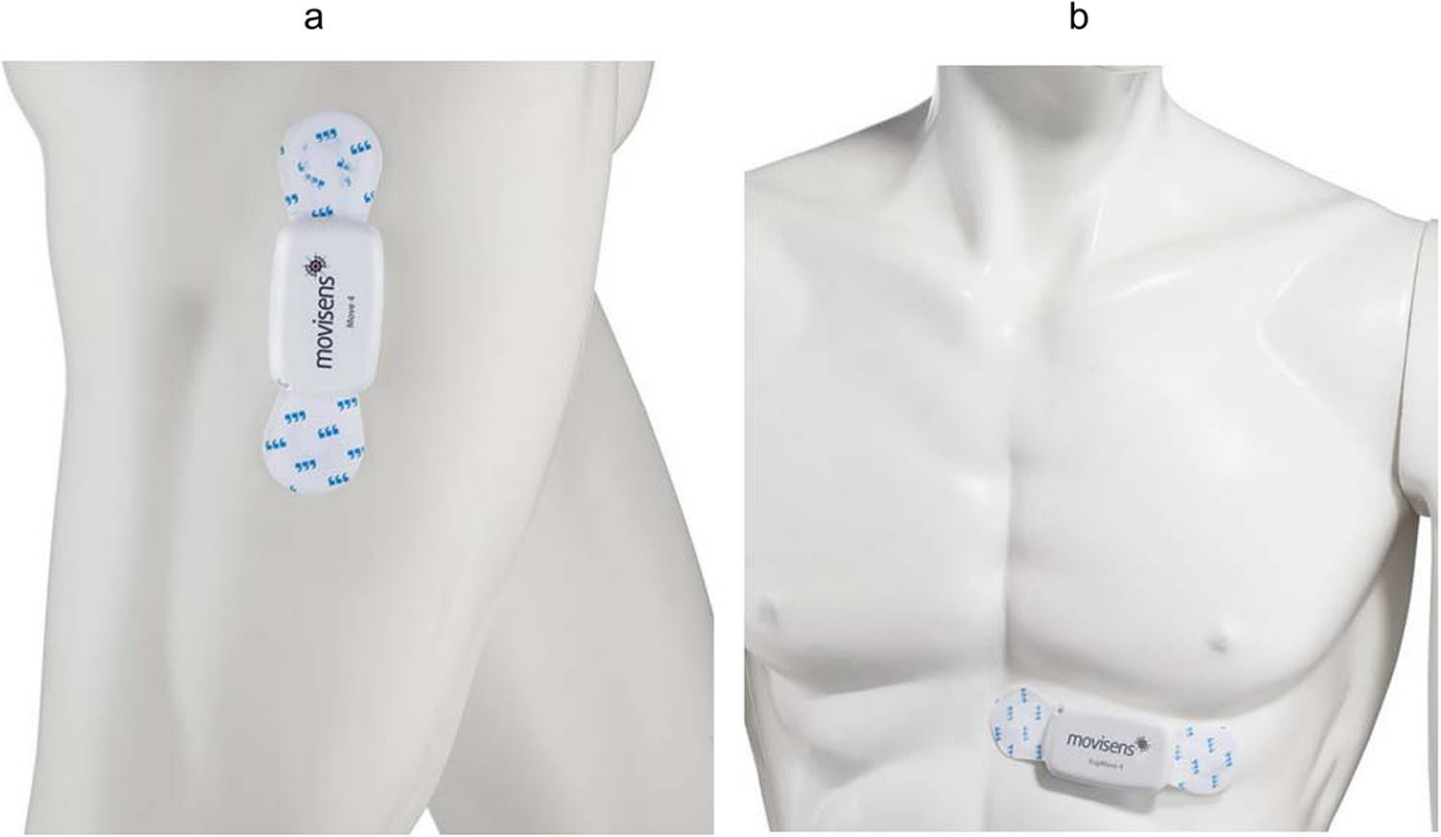
**a** Move 4 **b** ECG Move 4

The Move 4 [30] and ECG Move 4 [31] activity sensors were developed by movisens GmbH (Germany) and have been validated concerning step count (mean percentage deviation 0.6%) [32] and activity classes (accuracy 98.2%) [33]. The sensors are waterproof, the battery life is at least 3 days, they have a size of 62.3mm x 38.6mm x 11.5mm and weigh 25–26g (for retail prices see appendix 3). Integrated sensors (acceleration, rotation, pressure, temperature) continuously record raw physical data, the ECG Move 4 also includes an ECG sensor. Sensor configuration was done by the software "Sensor Manager" [34]. The sensors were attached with adhesive electrodes, the Move 4 vertically on the lateral right thigh, the ECG Move 4 horizontally from the xiphoid to the left chest (see Fig. 1). Body hair was shaved beforehand. The electrodes were checked for tightness during the daily visit and replaced if necessary. The "Data Analyzer" calculates physiological parameters such as activity classes (lying, sitting, standing, walking), step count and cardiac parameters (heart rate, heart rate variability) from the raw data [35].

### Endpoints

Time on feet (objectively by Move 4, sum of activity classes standing + walking), number of steps (Move 4) and time out of bed (patient self-assessment) were measured to quantify the mobilization endpoint. All parameters refer to POD1–2 (0–24 hrs each) and POD3 (0–20 hrs).

Other endpoints were mobilization on POD0, pain & complications POD0–3, length of hospital stay, duration of MI consultations and validation by MITI & CLEAR. Questionnaires were used to record preoperative activity (IPAQ Short Form – International Physical Activity Score [36], pre-op), self-efficacy (GSE – General Self-Efficacy Scale [37], pre-op and POD3), activation (PAM13 – Patient Activation Measure [38], pre-op and POD3), anxiety & depression (HADS – Hospital Anxiety and Depression Scale [39], pre-op and POD3), daily functioning (Barthel Index, pre-op and POD3), recovery (QoR-15 – Quality of Recovery [40], pre-op and POD1/3/28–32), and perceived empathy (REM – Rating Scale for the Assessment of Empathic Communication in Medical Interviews [41], POD3). Minimum and maximum achievable score GSE (0–40), PAM13 (13–52), HADS (0–21), Barthel (0–100), QoR-15 (0–150), REM (7–63).

The postulated successful blinding of the study participants was queried in a written form.

### Statistical analysis

Only complete data sets were included for the statistical analysis of each day. When comparing cumulative sum scores from PODs1–3, only patients with complete records on all three days were considered.

For group comparisons, the χ2-square test and Fisher's exact test (if requirements for χ2-square test not met) were used for nominal-scaled characteristics, and the Cochran-Armitage trend test for ordinal-scaled characteristics. For metric-scaled characteristics, the independent-samples t-test was used when data were approximately normally distributed; if not, the Wilcoxon-Mann-Whitney-Test was used. If the t-test was used, mean ± standard deviation were reported, if the Wilcoxon-Mann-Whitney-Test was used, median [quartile range = Q1-Q3]. A p-value < 0.05 (two-sided) is considered statistically significant. SAS® software 9.4 (SAS Institute, Inc., USA) was used for statistical analyses.

## Results

### Study population and design

**Fig. 2:**
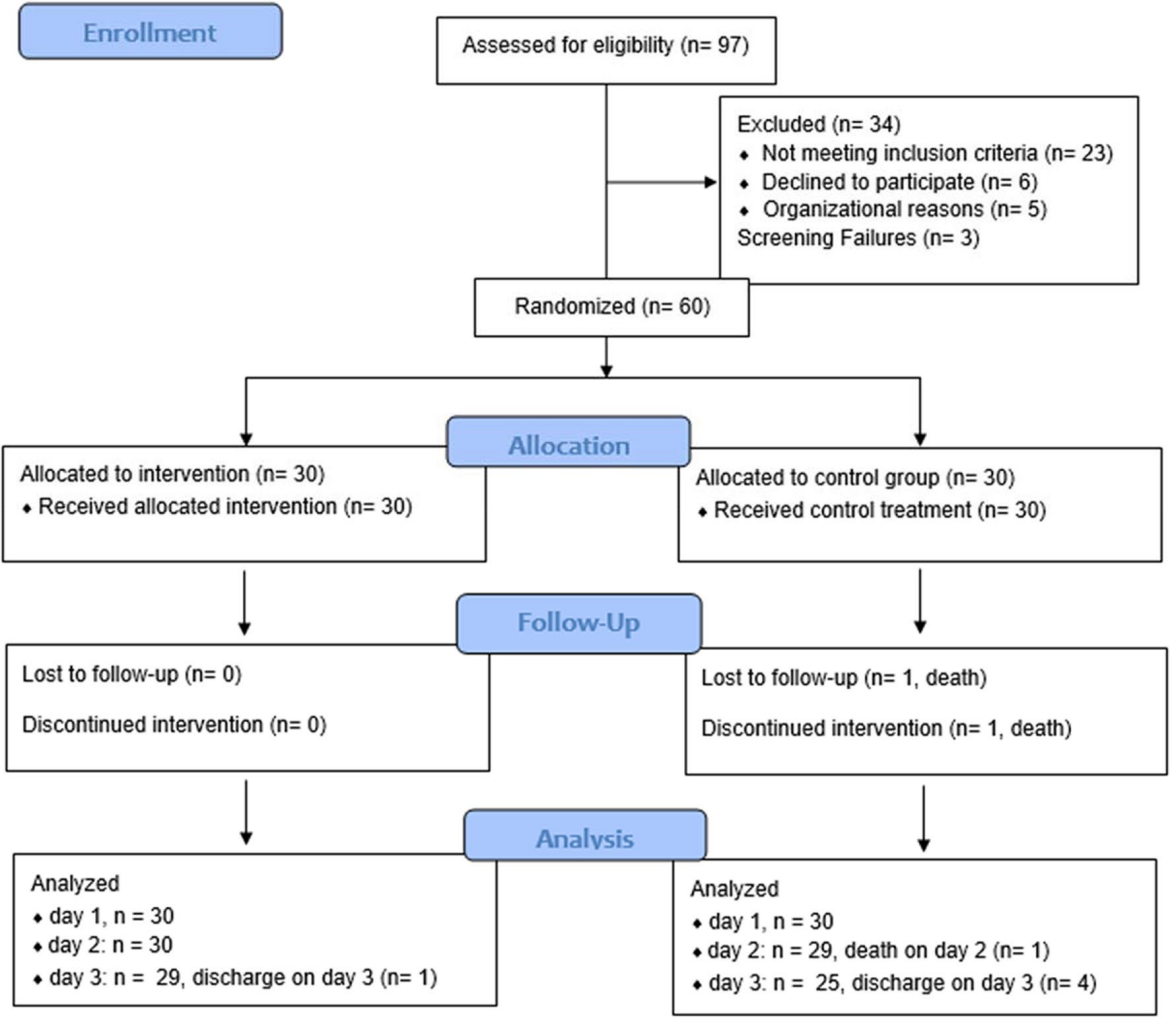
Consort flow diagram

Between March 2022 and July 2022 97 patients were screened and 60 participated in the final study (see CONSORT-Chart in Fig. 2). Originally, only 50 study participants were planned as pilot-study sample size, but due to incomplete data sets, the sample size was increased to 60 during the recruitment period (approved by the ethics committee). According to intention-to-treat principle all 60 randomized participants were included in the statistical analyses. The following deviations exist: The self-assessment "time out of bed" was collected from POD3 by subjects 11 and 12 onwards. Since only fully completed questionnaires were analyzed, the following sample sizes result: The sample size of pre-op and POD1 questionnaires was 60 (n_int_ & n_con_ = 30 each), except GSE pre-op (n_int_ = 27 & n_con_ = 26). Sample size of the POD3 and POD28–32 questionnaires was 59 (n_int_ = 30 & n_con_ = 29), except REM (n_int_ = 30 & n_con_ = 28). Since item 7 of the QoR-15 questionnaire ("getting support from hospital doctors and nurses") was completed by only 52 of 60 participants preoperatively and by none at POD28–32, the missing values were estimated with linear regression (pre-op using POD1 and post-op 28-32 using POD3).

All study participants were compliant with the protocol requirements and wore the motion sensors throughout day and night. Since the Move 4 fell of a few times during the initial phase, it was additionally covered with an adhesive film.

Study participants had an average age of 60.7 ± 13.3 years (range = 30-82y.), BMI of 26.4 ± 5, and 56.67% were male. 75% of the subjects were classified ASA II, 21.67% ASA III, and 3.33% ASA I. The mean duration of surgery was 216.7 ± 110.8 minutes, and 35% received a stoma during surgery. There were no significant differences between groups regarding all sociodemographic and clinical baseline data listed in Table 2.

**Table 2:** Baseline characteristics of study participants

	Intervention	Control	*p*-value
Gender (m/f)	15/15	19/11	0.297
Age*	62.2 ± 13.8	59.1 ± 12.9	0.378
BMI*	25.6 ± 4.6	27.3 ± 5.3	0.2
ASA (I/II/III)	1/22/7	1/23/6	0.781
Duration of surgery in min**	199.5 [146-264]	202 [123-306]	0.564
Stoma creation (yes/no)	9/21	12/18	0.417
Blood loss during op in ml**	100 [0-300]	0 [0-150]	0.123
Complications during OP (yes/no)	0/30	1/29	1
Pre-op bowel preparation (yes/no)	19/11	17/13	0.598
Pre-op anemia (yes/no)	10/20	10/20	1
Diabetes (yes/no)	4/26	3/27	1
Alcohol abuse (yes/no)	3/27	4/26	1
Smoking (yes/no)	5/25	6/24	0.739
Pre-op immunosuppression (yes/no)	2/28	1/29	1
Pre-op chemotherapy (yes/no)	1/29	5/25	0.195
ECOG status (0/1)	14/16	16/14	0.606
Walking aid before surgery (yes/no)	1/29	1/29	1
Pre-op questionnaires:			
MET absolute – IPAQ**	4607 [2373-7119]	3971 [2079-6678]	0.535
MET-level – IPAQ (high/medium/low)	20/9/1	19/9/2	0.658
Daily functioning – Barthel-Index**	95 [90-100]	100 [90-100]	0.391
Patient activation – PAM 13**	45 [41-48]	44 [40-47]	0.386
Anxiety – HADS**	6 [3-8]	6 [4-8]	0.656
Depression – HADS**	4 [2-7]	4 [3-7]	0.829
Self-efficacy – GSE**	30 [27-35]	33 [29-36]	0.432
Baseline Recovery – QoR-15**	132 [119-141]	126 [112-139]	0.332

* t-test: Mean ± SD; ** Wilcoxon-Mann-Whitney-test: Median (Q1-Q3). *BMI* body mass index, *ASA* American Society of Anesthesiology, *ECOG* Eastern Co-operative Oncology Group, *MET* metabolic equivalent, *IPAQ* International Physical Activity Score

Questionnaires (minimum – maximum achievable score): *Barthel-Index* (0-100); *PAM13* – Patient Activation Measure (13-52); *HADS* – Hospital Anxiety and Depression Scale (0-21); *GSE* – General Self-Efficacy Scale (0-40); *QoR-15* – Quality of Recovery (0-150)

The two most common surgical indications were malignant neoplasms (46.67%) and stoma-reversal / restoration of bowel continuity after Hartmann’s Procedure (35%) (see Table 3). 63.33% of the procedures were minimal-invasive (laparoscopic or daVinci), 33.33% were primarily open, and 3.33% were converted. Apart from one procedure (pelvic tumor debulking after rectal carcinoma), bowel resection was performed in all other operations (see Table 3).

**Table 3:** Op Indication & Surgery (*n* = 60)

	Intervention	Control
Op-indication		
Malignant disease	11	17
Crohn/ulcerative colitis	0	2
Diverticulitis	3	0
Stoma-Rev.&Hartmann	12	9
Rectal prolaps	2	1
Others	2	1
Type of Surgery		
Colonic resection	5	7
Rectal resection	9	10
Stoma revision	12	9
Others	4	4
Type of Intervention		
primary open	10	10
converted	1	1
laparoskopic	14	10
robotic assisted (daVinci)	5	9

### Mobilization endpoint

#### Time out of bed (Table 4)

The IG was significantly longer out of bed than the CG cumulatively during POD1–3; Median = 685 vs. 420 min, *p* = 0.022. The cumulative range (min-max) was 135-2675 min in the IG and 37-1110 min in the CG. When comparing individual days, differences were significant on POD1 and POD3, but not on POD2. The ERAS**®**-compliance goal of 360 minutes was achieved significantly more often in the IG across POD1–3, by 27.4% vs 10.61% in the CG (*p* = 0.013).

**Table 4:** Time out of bed

		Intervention	Control	*p*-value
Minutes Out of bed *Median [Q1-Q3]*	POD1 (n_int_=24, n_con_=24)*	217.5 [57.5-360]	90 [32.5-180]	0.037
	POD2 (n_int_=24, n_con_=23)*	240 [145-350]	180 [70-300]	0.198
	POD3 (n_int_=25, n_con_=19)*	240 [150-360]	165 [75-240]	0.047
	∑POD1-3 (n_int_=23, n_con_=19)*	685 [430-840]	420 [155-700]	0.022
ERAS**®**- mobilization goal reached (≥ 360 min per day) *n [%]*	POD1 (n_int_=24, n_con_=24)**	7 [29.17%]	1 [4.17%]	0.048
	POD2 (n_int_=24, n_con_=23)**	6 [25.00%]	4 [17.39%]	0.724
	POD3 (n_int_=25, n_con_=19)**	7 [28.00%]	2 [10.53%]	0.26
	∑POD1-3 (n_int_=73, n_con_=66)***	20 [27.4%]	7 [10.61%]	0.013

* Wilcoxon-Mann-Whitney-test, ** Fisher’s exact test, *** χ2-square test. POD = postoperative day; n_int_ = sample size intervention group; n_con_ = sample size control group

#### Time on feet and step count (Table 5)

Concerning the time on feet (activity class “standing” + “walking”) and step count measured by Move 4, the IG reached higher values every post-op day without reaching statistical significance.

**Table 5:** Time on feet & Step count

	Measured by Move 4	Intervention Median [Q1 – Q3]	Control Median [Q1 – Q3]	*p*-value*
Time on feet	POD1 (n_int_=30, n_con_=30)	95.5 [30-194]	67.5 [22-146]	0.322
	POD2 (n_int_=30, n_con_=29)	149.5 [61-219]	115 [54-168]	0.231
	POD3 (n_int_=29, n_con_=25)	138 [96-203]	113 [78-158]	0.187
	∑ POD1-3 (n_int_=29, n_con_=25)	384 [176-521]	267 [169-413]	0.212
	⌀ per POD (n_int_=30, n_con_=30)	131.5 [58.7-217.5]	95.8 [56.3-163]	0.179
Step count	POD1 (n_int_=30, n_con_=30)	646 [106-2089]	336 [31-2350]	0.473
	POD2 (n_int_=30, n_con_=29)	1882 [186-3372]	1030 [263-2941]	0.756
	POD3 (n_int_=29, n_con_=25)	1783 [620-3555]	1030 [443-2497]	0.252
	∑ POD1-3 (n_int_=29, n_con_=25)	3093 [1321-9084]	2180 [870-5955]	0.298
	⌀ per POD (n_int_=30, n_con_=30)	1347 [440-3360]	754 [290-2738]	0.333

* Wilcoxon-Mann-Whitney-test. POD = postoperative day; n_int_ = sample size intervention group; n_con_ = sample size control group. Step count rounded to whole steps

### Other endpoints

The length of postoperative hospital stay did not differ between groups (*p* = 0.546); the median [range] was 5 days [3-12] in the intervention and 5 days [3-20] in the CG. At POD0, 63% of the IG and 47% of the CG got up a first time (*p* = 0.195). From POD0–POD3, there were 3 complications in the IG (Clavien-Dindo: 2x grade I, 1x grade III), and 5 in the CG (Clavien-Dindo: 2x grade I, 2x grade II, 1x grade V), *p* = 0.707. Regarding maximum pain/day and average pain/day, the groups did not differ.

In the Quality of Recovery questionnaire, the IG achieved on POD1 a significantly better median [Q1-Q3] score of 115.5 [101-131] vs. CG 100 [83-113] (*p* = 0.049). There were no significant differences on POD3 and POD28–32. There were no significant differences between groups on any other postoperative questionnaire. Regarding subjectively perceived empathy, both groups had a median score of 63 (≙ maximum) in the REM questionnaire (*p* = 0.523). The median [Q1-Q3] / [range] duration of a MI conversation was 18.5 min [16-25] / [10-32] preoperatively and 11.88 min [10.75-14.75] / [8.75-22.75] postoperatively (POD0–3) (see appendix 4).

The detailed validation results of the MI sessions by the MITI and the CLEAR instrument can be seen in appendix 5. In the four global rating categories, on a Likert scale of 1 (minimum MI fidelity) to 5 (maximum MI fidelity), an average score of 3.83 (interviewer 1) and 3.79 (interviewer 2) was achieved in the IG. In the CG interview, the average score was less than half for both interviewers (1.75). There were no differences between the two conducting MI interviewer concerning the mobilization endpoints. 19 of 29 study participants in the CG suspected on POD3 that they were in the IG (with MI), 7 others were unsure, and only 3 subjects correctly defined their group assignment (control) indicating a successful patient-blinding (see appendix 6).

## Discussion

The data from this pilot-study provide preliminary evidence that MI can improve mobilization in the setting of ERAS**®**-certified elective bowel surgery. It was shown that with MI, a significantly longer time was spent out of bed cumulatively over POD1–3. On all three PODs, the IG spent a median of over one hour longer out of bed than the CG, on POD1 it was even over two hours. In addition, the IG achieved significantly more often the ERAS**®**-compliance goal across PODs 1–3. 

 It should be noted, however, that despite significantly better performance, only 27.4% of the IG achieved the ERAS**®**-compliance goal of 360 min/day. This finding is in line with the prospective data analysis of Gustafsson et al., in which also only about a quarter of the patients reached the ERAS**®** daily mobilization target [11]. Our results provide suggestions to discuss the usefulness and feasibility of general ERAS**®** mobilization goals in follow-up studies.

Although there was no significant difference between the groups in the other endpoints for mobilization (time on feet and step count), the IG was able to achieve a higher median for all parameters (on each individual POD and cumulatively). Thus, a tendency is emerging that MI probably positively influence activity in addition to the ERAS**®**-target "time out of bed".

In another ERAS**®** abdominal surgery study, the study group with a significantly effective intervention (activity boards with elaborated goals) spent a median of 78 minutes/day on their feet and had a step count of 1057 steps/day via POD1–POD3 [42]. In the present study MI achieved higher results, with a median of 131.5 minutes/day (time on feet) and 1347 steps/day. Furthermore, compared to another colorectal surgery study, our MI group achieved higher step counts than the IG of a significantly effective staff-assisted mobilization program (comparison of medians; POD1: 646 vs 542, POD2: 1882 vs 1021, POD3: 1783 vs 521) [43]. Interestingly, despite a lower number of steps, the subjects of the mobilization program spent more time out of bed in self-report than our MI group (comparison of medians; POD1: 3.63h vs. 7h, POD2: 4h vs. 6h, POD3: 4h vs. 6h). This demonstrates the difference between objective measurements and subjective feelings about mobilization.

Other postoperative mobilization programs have also shown positive effects [44, 45], but the increased activity was mainly performed while under the care of nurses or physiotherapists and was obtained by a large amount of staff resources. Because MI primarily enhances intrinsic motivation [14], patients are more likely to mobilize on their own initiative and less dependent on staff. Therefore, time requirement for MI can be considered low compared to staff-assisted mobilization programs. Other effective interventions are fitness trackers [46, 47] and educational videos on the advantages & disadvantages of mobilization [48]. Especially regarding fitness trackers, increasing research is being conducted, such as in the EXPELLIARMUS-Trial [49]. EXPELLIARMUS investigates whether fitness tracker-based feedback can reduce complications after major abdominal surgery. A combination of several mobilization-promoting interventions could have additional effect and should be investigated.

The MI intervention could be well integrated into the daily ward routine. With a median duration of 18.5 minutes preoperatively and 11.88 minutes postoperatively, MI is low-threshold and the basic knowledge can be learned quickly in a three-day course. In the MI evaluations by MITI and CLEAR, moderate to high validity was achieved. While the MITI score for empathy turned out to be clearly higher in the IG than in the control (4 vs. 1), no difference was found in the subjective patient-assessment in the REM-questionnaire. Since in both groups the REM-median was at the maximum score, there might have been no discrimination between the groups due to the ceiling-effect. Nevertheless, the non-significant result in the REM-questionnaire fits the evidence, that the single blinding of the study participants worked. Only 3 subjects in the CG had correctly guessed their study group, 7 were unsure, and 19 even thought they had been in the IG. 

The self-created guideline for the MI conversations (see appendix 2) proved to be a very helpful orientation. Since the guide was used in all 6 MI interviews evaluated by MITI, the partial standardization does not seem to have a negative impact on validity. The guide provides a rough insight into the MI-conversations, in contrast to most MI-studies with the mere statement "we did MI.". Such guides can be helpful in conceptualizing follow-up studies and provide guidance during implementations in practice. This pilot-study is one of the first papers examining MI to increase mobilization in perioperative medicine. Taylor et al. published a study protocol for a RCT testing whether telephone-based MI sessions can increase walking time in ≥65-year-olds after hip fracture [50]. Enrollment was completed in June 2022, follow-up was planned up to mid-2023. To our current knowledge, the present work is also the first study to investigate MI in the context of ERAS**®** surgery. Subsequent work will examine whether MI can also positively influence other ERAS**®** treatment recommendations with poor adherence [4, 9], such as daily protein-supplementation and calory-intake goals.

The present pilot-study has limitations. It was not possible to measure "time out of bed" completely objectively by the sum of the activity classes walking + standing + sitting, because time periods were also measured as "sitting” when the bed head was elevated. Therefore, recording by self-assessment was started from the eleventh study participant onwards, which was the main reason for a reduced sample size together with early discharges on POD3. There were more early discharges on POD3 in the CG than in the intervention (4 vs. 1). It could be argued that in the control, a larger number of fit patients (because they could already go home on POD3) were not included in the cumulative analysis of POD1–3. However, when comparing POD1 with POD3 for "time out of bed", the differences at POD1 had a smaller p-value (*p* = 0.037 vs. p = 0.047). When comparing individual PODs and in comparison to other studies, it is important to note that all endpoints were only measured until 8 pm at POD3. No data were collected for a possible sustained MI-effect beyond hospital discharge; for example, a mobilization program that was effective in the hospital had no longer a mobilization-enhancing effect 4 weeks post-op [43]. Despite many measures to ensure high MI fidelity (basic course, supervision, guideline & validations), MI validation occurred only in a few visits and cannot be generalized to all visits due to the individuality of a MI session.

## Conclusion

The present pilot-study generated first evidence that Motivational Interviewing can improve postoperative mobilization in the context of ERAS**®** bowel surgery. The MI intervention was well integrated into the daily routine of the ward and could be carried out with a moderate extra time effort of median 12 minutes in the postoperative visits. This study provides first indications for practical implications. It might be useful to train staff members of all care levels in MI (physicians, nurses, physiotherapists, etc.), who are involved in the ERAS**®** treatment pathway. The findings and design of this pilot-study provides a model and rationale to further evaluate the beneficial effect of Motivational Interviewing within the perioperative continuum by a multicenter follow-up trial.

### Supplementary information

ESM 1 (PDF 379 KB)

## Declarations

### Publication history

The submitted manuscript has not yet been published. Only an abstract of the paper is available online as part of the ERAS World Congress (https://erascongress.com/program/2023/OR04.cgi) and the German Surgical Congress (https://dck2023.de/einfluss-von-motivational-interviewing-auf-die-postoperative-mobilisation-im-enhanced-recovery-after-surgery-eras-klinikpfad-bei-elektiver-darmchirurgie-eine-randomisiert-verblindete-pilotstudie/).

## References

[1] Ljungqvist O, Scott M, Fearon KC (2017). Enhanced recovery after surgery: a review. JAMA Surg.

[2] Ljungqvist O, de Boer HD, Balfour A, Fawcett WJ, Lobo DN, Nelson G (2021). Opportunities and challenges for the next phase of enhanced recovery after surgery: a review. JAMA Surg.

[3] Schwenk W (2021). Enhanced recovery after surgery-Does the ERAS concept keep its promises. Chirurg.

[4] Ripolles-Melchor J, Ramirez-Rodriguez JM, Casans-Frances R, Aldecoa C, Abad-Motos A, Logrono-Egea M (2019). Association between use of enhanced recovery after surgery protocol and postoperative complications in colorectal surgery: the postoperative outcomes within enhanced recovery after surgery protocol (power) study. JAMA Surg.

[5] Greer NL, Gunnar WP, Dahm P, Lee AE, MacDonald R, Shaukat A (2018). Enhanced Recovery Protocols for Adults Undergoing Colorectal Surgery: A Systematic Review and Meta-analysis. Dis Colon Rectum.

[6] Gustafsson UO, Scott MJ, Hubner M, Nygren J, Demartines N, Francis N (2019). Guidelines for perioperative care in elective colorectal surgery: enhanced recovery after surgery (eras((r))) society recommendations: 2018. World J Surg.

[7] Kehlet H (2018). ERAS implementation-time to move forward. Ann Surg.

[8] Turan A, Khanna AK, Brooker J, Saha AK, Clark CJ, Samant A et al (2023) Association between mobilization and composite postoperative complications following major elective surgery. JAMA Surgery. 10.1001/jamasurg.2023.1122 10.1001/jamasurg.2023.1122 PMC10233451 37256591

[9] van Zelm R, Coeckelberghs E, Sermeus W, Buck D, van Overstraeten A, Weimann A, Seys D (2017). Variation in care for surgical patients with colorectal cancer: protocol adherence in 12 European hospitals. Int J Colorectal Dis.

[10] Grass F, Pache B, Martin D, Addor V, Hahnloser D, Demartines N (2018). Feasibility of early postoperative mobilisation after colorectal surgery: a retrospective cohort study. Int J Surg.

[11] Gustafsson UO, Hausel J, Thorell A, Ljungqvist O, Soop M, Nygren J (2011). Adherence to the enhanced recovery after surgery protocol and outcomes after colorectal cancer surgery. Arch Surg.

[12] Miller WR, Rollnick S (1991). Motivational interviewing: preparing people to change addictive behavior. Motivational interviewing: preparing people to change addictive behavior.

[13] Miller WR, Rollnick S (2015). Motivational interviewing. 3. Auflage des Standardwerks in.

[14] Csillik A (2014). Positive motivational interviewing: activating clients’ strengths and intrinsic motivation to change. J Contemp Psychother.

[15] Soderlund LL, Madson MB, Rubak S, Nilsen P (2011). A systematic review of motivational interviewing training for general health care practitioners. Patient Educ Couns.

[16] Jacobs NN, Calvo L, Dieringer A, Hall A, Danko R (2021). Motivational interviewing training: a case-based curriculum for preclinical medical students. MedEdPORTAL.

[17] Smedslund G, Berg RC, Hammerstrom KT, Steiro A, Leiknes KA, Dahl HM (2011). Motivational interviewing for substance abuse. Cochrane Database Syst Rev.

[18] Lundahl B, Moleni T, Burke BL, Butters R, Tollefson D, Butler C (2013). Motivational interviewing in medical care settings: a systematic review and meta-analysis of randomized controlled trials. Patient Educ Couns.

[19] Moitra E, Frost H, Campbell P, Maxwell M, O’Carroll RE, Dombrowski SU et al (2018) Effectiveness of motivational interviewing on adult behaviour change in health and social care settings: a systematic review of reviews. Plos One 13(10). 10.1371/journal.pone.0204890 10.1371/journal.pone.0204890 PMC6193639 30335780

[20] Nuss K, Moore K, Nelson T, Li K (2021). Effects of motivational interviewing and wearable fitness trackers on motivation and physical activity: a systematic review. Am J Health Promot.

[21] Wade M, Brown N, Steele J, Mann S, Dancy B, Winter S (2021). The impact of signposting and group support pathways on a community-based physical activity intervention grounded in motivational interviewing. J Public Health (Oxf).

[22] David LA, Sockalingam S, Wnuk S, Cassin SE (2016). A pilot randomized controlled trial examining the feasibility, acceptability, and efficacy of adapted motivational interviewing for post-operative bariatric surgery patients. Eat Behav.

[23] Hah JM, Trafton JA, Narasimhan B, Krishnamurthy P, Hilmoe H, Sharifzadeh Y (2020). Efficacy of motivational-interviewing and guided opioid tapering support for patients undergoing orthopedic surgery (MI-Opioid Taper): a prospective, assessor-blind, randomized controlled pilot trial. EClinicalMedicine.

[24] Skolasky RL, Maggard AM, Wegener ST, Riley LH (2018). Telephone-based intervention to improve rehabilitation engagement after spinal stenosis surgery: a prospective lagged controlled trial. J Bone Joint Surg Am.

[25] Seyfried S, Herrle F, Schroter M, Hardt J, Betzler A, Rahbari NN (2021). Initial experiences with the implementation of the enhanced recovery after surgery (ERAS(R)) protocol. Chirurg.

[26] GK Quest Akademie - Grundkurs Motivational Interviewing (MI). https://www.gk-quest.de/Seminare/Seminarthemen/Termine/grund/33 Accessed 17.11.2023.

[27] GK Quest Akademie - Team für Motivational Interviewing (MI). https://www.motivational-interview.de/wer-wir-sind/unser-team Accessed 17.11.2023.

[28] Moyers TB, Rowell LN, Manuel JK, Ernst D, Houck JM (2016). The motivational interviewing treatment integrity code (MITI 4): rationale, preliminary reliability and validity. J Subst Abuse Treat.

[29] Glynn LH, Moyers TB (2012) Manual for the Client Language EAsy Rating (CLEAR) Coding System: Formerly “Motivational Interviewing Skill Code (MISC) 1.1". https://citeseerx.ist.psu.edu/document?repid=rep1&type=pdf&doi=a0025cd9c936f148d7301379614274d4e7d076ff. Accessed 17.11.2023.

[30] movisens - Move 4 Aktivitätssensor https://www.movisens.com/de/produkte/aktivitaetssensor/ Accessed 17.11.2023.

[31] movisens - EcgMove 4 - EKG- und Aktivitätssensor. https://www.movisens.com/de/produkte/ekg-sensor/ Accessed 17.11.2023.

[32] Anastasopoulou P, Härtel S, Hey S (2013). A comparison of two commercial activity monitors for measuring step counts during different everyday life walking activities. Int J Sports Sci Eng.

[33] Anastasopoulou P, Tansella M, Stumpp J, Shammas L, Hey S (2012). Classification of human physical activity and energy expenditure estimation by accelerometry and barometry. Annu Int Conf IEEE Eng Med Biol Soc.

[34] movisens - SensorManager. https://www.movisens.com/de/produkte/sensormanager/ Accessed 17.11.2023.

[35] movisens - DataAnalyzer – Analyse der Sensor-Daten. https://www.movisens.com/de/produkte/dataanalyzer/ Accessed 17.11.2023.

[36] Craig CL, Marshall AL, Sjostrom M, Bauman AE, Booth ML, Ainsworth BE (2003). International physical activity questionnaire: 12-country reliability and validity. Med Sci Sports Exerc.

[37] Schwarzer R (2012) The general self-efficacy scale (GSE).1-4.

[38] Hibbard JH, Mahoney ER, Stockard J, Tusler M (2005). Development and testing of a short form of the patient activation measure. Health Serv Res.

[39] Zigmond AS, Snaith RP (1983). The hospital anxiety and depression scale. Acta Psychiatrica Scandinavica.

[40] Stark PA, Myles PS, Burke JA (2013). Development and psychometric evaluation of a postoperative quality of recovery score: the QoR-15. Anesthesiology.

[41] Nicolai J, Demmel R, Hagen J (2007). Rating Scales for the assessment of empathic communication in medical interviews (REM): scale development, reliability, and validity. J Clin Psychol Med Settings.

[42] Porserud A, Aly M, Nygren-Bonnier M, Hagströmer M (2019). Objectively measured mobilisation is enhanced by a new behaviour support tool in patients undergoing abdominal cancer surgery. Eur J Surg Oncol.

[43] Fiore JF, Castelino T, Pecorelli N, Niculiseanu P, Balvardi S, Hershorn O (2017). Ensuring early mobilization within an enhanced recovery program for colorectal surgery: a randomized controlled trial. Ann Surg.

[44] de Almeida EPM, de Almeida JP, Landoni G, Galas F, Fukushima JT, Fominskiy E (2017). Early mobilization programme improves functional capacity after major abdominal cancer surgery: a randomized controlled trial. Br J Anaesth.

[45] Ni CY, Wang ZH, Huang ZP, Zhou H, Fu LJ, Cai H (2018). Early enforced mobilization after liver resection: a prospective randomized controlled trial. Int J Surg.

[46] de Leeuwerk ME, Bor P, van der Ploeg HP, de Groot V, van der Schaaf M, van der Leeden M (2022). The effectiveness of physical activity interventions using activity trackers during or after inpatient care: a systematic review and meta-analysis of randomized controlled trials. Int J Behav Nutr Phys Act.

[47] Wolk S, Linke S, Bogner A, Sturm D, Meissner T, Mussle B (2019). Use of activity tracking in major visceral surgery-the enhanced perioperative mobilization trial: a randomized controlled trial. J Gastrointest Surg.

[48] Jones ASK, Kleinstauber M, Akroyd A, Mittendorf A, Bognuda P, Merrie AEH (2019). Using animated visualization to improve postoperative mobilization: a randomized controlled trial. Health Psychol.

[49] Schwab M, Brindl N, Studier-Fischer A, Tu T, Gsenger J, Pilgrim M (2020). Postoperative complications and mobilisation following major abdominal surgery with vs. without fitness tracker-based feedback (EXPELLIARMUS): study protocol for a student-led multicentre randomised controlled trial (CHIR-Net SIGMA study group). Trials.

[50] Taylor NF, O'Halloran PD, Watts JJ, Morris R, Peiris CL, Porter J (2021). Motivational interviewing with community-dwelling older adults after hip fracture (MIHip): protocol for a randomised controlled trial. BMJ Open.

