# Supplementary material for: Influence of motivational interviewing on postoperative mobilization in the enhanced recovery after surgery (ERAS®) pathway in elective colorectal surgery - a randomized patient-blinded pilot study
Source: Langenbecks Arch Surg. 2024 Apr 22;409(1):134. doi: 10.1007/s00423-024-03321-z (PMC11033226; doi:10.1007/s00423-024-03321-z)
Supplement: Supplementary file 1 — (PDF 379 KB) [file 423_2024_3321_MOESM1_ESM.pdf]

Influence of Motivational Interviewing on postoperative mobilization in the Enhanced Recovery after Surgery (ERAS®) Pathway in elective colorectal surgery - a randomized patient-blinded pilot study *(published in Langenbeck's Archives of Surgery)*

Rico Wiesenberger, B.Sc. [1]; Julian Müller [1]; Mario Kaufmann [1]; Christel Weiß, Prof. Dr. [2]; David Ghezel-Ahmadi, Dr. [3]; Julia Hardt, Prof. Dr. [1]; Christoph Reissfelder, Prof. Dr. [1, 4]; Florian Herrle, PD Dr. [1]

[1]Department of Surgery, Universitätsmedizin Mannheim, Medical Faculty Mannheim, Heidelberg University, Theodor-Kutzer-Ufer 1-3, 68167, Mannheim, Germany

[2]Institute for Medical Statistics, Universitätsmedizin Mannheim, Medical Faculty Mannheim, Heidelberg University, Theodor-Kutzer-Ufer 1-3, 68167, Mannheim, Germany

[3]Department of Anaesthesiology and Critical Care Medicine, Universitätsmedizin Mannheim, Medical Faculty Mannheim, Heidelberg University, Theodor-Kutzer-Ufer 1-3, 68167, Mannheim, Germany

[4]DKFZ-Hector Cancer Institute at the University Medical Center Mannheim, Germany.

Corresponding author:

PD Dr. Florian Herrle

Department of Surgery, Universitätsmedizin Mannheim

Theodor-Kutzer Ufer 1-3, 68167 Mannheim, Germany

fherrle@googlemail.com

Appendix

1. Example MI-conversation

In the following, an exemplary MI conversation from the preoperative visit is presented. All five core competencies of MI conversation (open questions, active listening, appreciation, summarize, offering advice) were used here. The core competency used is noted in parentheses after each of the MI doctoral student's statements. In addition, the number of the element used (E.) from the preoperative MI guideline (see Box 1) is given, if one of the elements was used.

Clinical background information (medical record): 52-year-old patient, ASA 2, ECOG 0, with sigmoid carcinoma planned for elective laparoscopic sigmoid resection in 3 days. Except of grade I arterial hypertension and a history of appendectomy 10 years ago, the patient has no relevant other disease or previous surgery.

MI doctoral student: "Thank you very much for participating in the study and for being willing to wear the sensors. It is not a matter of course!" (Appreciation; E. 1)

- Patient: "You're welcome!"

"Now it's three days until surgery. How are you doing today?" (Open question; E. 2)

- "I would say okay under the circumstances. I'm a little bit nervous."

"The major surgery is a new situation for you, and you don't know exactly what to expect." (Active listening)

- "Yes exactly. Except of a small appendectomy, I haven't had any surgery. I've always been pretty healthy, and now this..."

"The diagnosis was a shock for you at first". (Active listening)

- "Yes, I have no symptoms at all! If there hadn't been blood in my stool, I wouldn't have noticed it until today. The diagnosis turned my life upside down."

"'You actually feel as healthy as you did before, and nevertheless, the diagnosis has completely changed your life.'" (Active listening)

- "I'm just very uncomfortable with knowing that there's something growing in my belly right now that doesn't belong there."

"You will be glad when the tumor is extracted on Monday". (Active listening)

- "Yes, definitely. I want it behind me."

[...]

"You had the ERAS consultation with our ERAS nurse earlier and probably got quite a lot of information. How was that conversation for you?" (Open-ended question; E. 3)

- "The conversation was good and the ERAS nurse was very friendly. I have a positive impression of the clinic and the ERAS program. I hope to be able to go home as soon as possible after surgery."

" You feel like you are in good hands. And want to quickly get fit again after the operation with ERAS." (Active listening)

- "That would be nice if that succeeds."

„What do you think you can contribute to getting fit again as quickly as possible after your operation?“ (Open question; E. 5)

- "I think it's important that I don't let myself down. In the ERAS consultation, it was said that I should eat early and get up. I will try to do that."

"You are willing to actively help with your recovery and want to achieve the goals of the ERAS program." (Active Listening)

- "Yes, I want to help and mobilize, it's about my health after all."

"On a scale of 0 (not at all important) to 10 (very important), how important is mobilization after surgery for you?" (Importance scale; E. 10)

- "I would say an 8. But the ERAS-brochure says something about 6h of activity a day, which is way too long in my opinion."

"You think it's pretty important to mobilize after surgery, but the ERAS goal of 6h seems unrealistically high to you" (Active listening).

- "Yes, I have to be honest about that. I don't even move that much at home!"

"If you would like, I can give you some more information about this?" (Offering advice)

- "Sure!"

"The 6h refers to time out of bed from the first day after surgery, so this includes sitting out of bed, for example at the table." (Giving advice after consent)

- "Oh well, I misunderstood that then. But for the day after the operation, it still seems too much to me."

"You can assess your own resources by yourself best!" (Emphasize autonomy)

"What goals do you consider realistic for yourself?" (Open question, E. 8)

- "I think 3-4 hours is what I could achieve."

"Very good, you are confident with 3.5 hours. That is still an ambitious goal!" (Active listening)

"How confident are you that you will achieve your self-set 3.5h goal after surgery, on a scale of 0 (not at all confident) to 10 (very confident)?" (Confidence scale, E. 11)

- "This is difficult to assess, from a gut feeling I would say a 9."

"The question is difficult for you to assess because you don't know yet how you will feel after surgery." (Active listening)

- "Exactly. As I said, it's the first major surgery for me, so I don't know yet how I'll feel after such a procedure."

"And despite this uncertainty, you are very confident with a 9 that you will achieve your mobilization goal." (Active listening)

- "Yes, when I set my mind to something, I want to achieve it. And most of the time I succeed."

"You have a strong fighting spirit!" (Active listening, appreciation)

[...]

"What are your plans to achieve those 3.5 hours out of bed?" (Open question, E. 9)

- "I'm going to split it up throughout the day. Go for a walk a few times a day. I also want to take a book with me, while reading I can sit down somewhere."

"You've got some good ideas already! If you like, I can give you another tip about what others have done in your situation?" (Offer advice)

- "Gladly."

"Some patients always ate their meals at the table in the hospital room, so they could collect additional minutes out of bed." (Giving advice after consent)

- "That is also a good idea! I will try to implement that."

"Great! To sort it all out a little bit, I would summarize the conversation again briefly, if you would?" (Asking for consent to summarize)

- "Sure."

"The diagnosis was a shock to you at first and has worried you a lot, but now you're glad when the surgery is over. After the operation, you said that your goal is to be fit for discharge as quickly as possible with ERAS and that you are also willing to actively help with this. You set yourself a mobilization goal of 3.5 hours and are very confident that you will achieve this goal. You are self-confident because you have often achieved the goals you set. Did I understand that correctly?" (Summary, E. 12)

- "Yes, you got that right." / [...]

"Good, what else is on your mind?" (Open question, E. 13)

- "Nothing really, so far I feel well prepared for the operation." / [...]

"All right. Thank you very much for the conversation. I think your attitude is great, that you don't let yourself be discouraged despite the diagnosis and now just want to solve this problem in a goal-oriented way. That's very valuable." (Appreciation, E.14)

"We'll see us after your surgery." (Farewell)

2. Guidelines for MI-consultation

The three boxes show the translated guidelines for three MI-consultations in our study (preoperative, postoperative day 0 & 1). As there were large individual differences in which elements of this guidelines were used in the interviews, the percentage use of each MI-element is given [ ]. The grey text passages belong to the nutrition part of the MINT-ERAS project (https://drks.de/search/en/trial/DRKS00027863).

Box 1: Guideline for preoperative MI-consultation

pre-op:

Date: Alias: Duration of visit: MI-Duration:

1. Appreciation – thanks for study participation ☐ [96,67%]
2. "Now you're here x days before surgery, how are you doing today?"☐ [93,33%]
3. „You just had the ERAS consultation and probably got a lot of information. How was the consultation for you?" ☐ [90%]
4. „Which aspects of the ERAS treatment pathway were especially important for you?" ☐ [60%]
5. „What do you think you can contribute to getting fit again as quickly as possible after your operation?" ☐ [16,67%]
6. „What do you already know about the mobilization/*nutrition* goals from the ERAS treatment pathway?" ☐ [50%]
7. „What do you think about the ERAS goals regarding mobilization/*nutrition*?" ☐ [46,67%]
8. „What goals do you consider realistic for yourself?" ☐ [33,33%]
9. „How do you think you can do that, that you can achieve those x hours out of the bed?" ☐ [30%]
10. „On a scale of 0 (not at all important) – 10 (very important), how important is the topic of mobilization/*nutrition* for you?" ☐ [96,67%]
11. „How confident are you about achieving the goals after surgery, on a scale of 0 (not at all confident) – 10 (very confident)?" ☐ [100%]

Conclusion:

12. Summary by the interviewer ☐ [86,67%]
13. Question about open concerns (e.g., "What is still on your mind?" or "What would you still like to talk about?") ☐ [83,33%]
14. Closing with appropriate appreciation ☐ [86,67%]

Box 2: Guideline for MI-consultation on day of surgery

post-op day 0:

Date: Alias: Duration of visit: MI-Duration:

1. „How are you doing now after overcoming the surgery?“ ☐ [100%]
2. „What have you done since you’ve arrived on the ward?“ ☐ [73,33%]
3. „What have you already ingested today?“ ☐ [43,33%]
4. „How have you already mobilized?“ ☐ [13,33%]
5. „If you think about the ERAS goals for mobilization *and nutrition* that we talked about before the operation. What do you still remember about that?“ ☐ [73,33%]
6. „If you look at the ERAS goals again: From the first post-op day 6h Out of bed*, 3 meals + 3 shakes*. What do you think about it now after surgery?“ ☐ [66,67%]
7. „What are your own goals?“ ☐ [30%]
8. „Before the operation, you had rated the importance of the topics mobilization*/nutrition* with x. And when I asked you about your confidence in achieving your goals, you said x. How confident are you now after surgery on a scale of 0-10 that you will achieve your goals tomorrow?“ ☐ [96,67%]
9. „What plan do you have to achieve your goal of spending x hours out of bed?“ ☐ [20%]

Conclusion:

10. Summary by the interviewer ☐ [66,67%]
11. Question about open concerns ☐ [80%]
12. Closing with appropriate appreciation ☐ [90%]

Box 3: Guideline for MI-consultation on postoperative day 1

post-op day 1:

Date: Alias: Duration of visit: MI-Duration:

1. „How are you today, one day after surgery?“ ☐ [96,67%]
2. „What have you done today?“ ☐ [90%]
3. Appreciation if appropriate in terms of behavior/attitude/performance ☐ [86,67%]
4. „What were you able to accomplish from the goals you set yesterday?“ ☐ [10%]
5. „How satisfied are you with your performance?“ ☐ [26,67%]
6. „What could be even better?“ ☐ [26,67%]
7. „What are your goals for tomorrow?“ ☐ [83,33%]

Conclusion:

8. Summary by the interviewer ☐ [53,33%]
9. Question about open concerns ☐ [93,33%]
10. Closing with appropriate appreciation ☐ [90%]

3: Retail prices Move 4 & ECG Move 4

The motion sensors used in this study were purchased from movisens GmbH. The retail prices depend on the number of devices and are between 550-600€ for the Move 4 and between 1800-2000€ for the ECG Move 4.

4: Duration of study visits

Duration in minutes	Intervention *MI only / MI + visit*	Control *visit*
Pre-op	18.5 [16-25] / 26* [21-33]	11.5* [9-16]
POD0	15.0 [10-19] / 35 [27-38]	20.5 [17-27]
POD1	12.0 [10-14] / 26.5 [23-32]	18.0 [15-21]
POD2	12.5 [11-16] / 28 [23-31]	17.0 [15-22]
POD3	9.0 [8-13] / 28 [23-33]	24.0 [18-30]

All results reported as Median [Q1-Q3] in minutes. *n ≤ 18, apart from this n ≙ consort diagram (figure 4). *MI* = Motivational Interviewing; *POD* = postoperative day.
MI + visit: Total time for intervention visit, i.e. MI + routine visit (equipment check, standard questions, etc.); MI only: Time spent talking to patients in MI style.

5: Motivational Interviewing (MI) validation by Motivational Interviewing Treatment Integrity & Client Language Easy Rating / Motivational Interviewing Skill Code (CLEAR / MISC)

		preop - JM	day 0 - JM	day 1 - JM	control (d1) - JM	preop - RW	day 0 - RW	day 1&2 - RW	control (d1) - RW
**Behavior counts** (MI-adherent)	Giving information (n)	3	2	2	11	6	6	9	6
	Structuring Statements (n)	0	3	4	13	3	3	5	10
	Question total (n)	5	6	5	38	12	11	22	33
	Open Question (OQ) (n)	3	5	5	3	7	8	14	5
	Closed Question (CQ) (n)	2	1	0	35	5	3	8	28
	Persuade with permission (n)	3	2	0	2	0	1	2	0
	Simple reflection (SR) (n)	14	17	14	7	14	16	18	1
	Complex reflection (CR) (n)	2	4	3	0	5	2	4	3
	Summary (n)	1	0	0	0	1	2	1	0
	Affirm (n)	4	4	4	0	2	2	6	1
	Seeking collaboration (n)	1	0	0	0	1	1	2	0
	Emphasizing autonomy (n)	1	1	0	0	1	0	0	0
(MI-Non-Adherent)	Persuade (n)	1	0	0	1	0	0	0	3
	Confront (n)	0	0	0	0	0	0	0	0
**Global Rating**	Partnership (1-5)	4	4	4	1	4	4	3	1
	Empathy (1-5)	4	4	4	1	4	4	4	1
	support change talk* (1-5)	4	4	3	1	3	4	4	1
	reduce sustain talk ** (1-5)	4	4	3	4	3,5	4	4	4
**Bench-marks**	Relational aspects (1-5)	4	4	4	1	4	4	3,5	1
	Technical aspects (1-5)	4	4	3	2,5	3,25	4	4	2,5
	%CR = CR/(SR+CR)	12,50%	19,05%	17,65%	0%	26,32%	11,11%	18,18%	75%
	R:Q = (SR+CR):(OQ+CQ)	3,2:1	3,5:1	3,4:1	0,2:1	1,6:1	1,6:1	1,0:1	0,1:1
**CLEAR/MISC**	Change Talk* (n)	16	17	14	10	20	21	17	7
	Sustain Talk ** (n)	2	5	6	1	4	3	10	11
	Neutral (n)	15	19	20	42	20	16	24	44

*, ** Adapted for our study: * positive comments about intervention, ** negative comments about intervention. d/day = postoperative day; JM / RW = initials of conducting interviewers

6: Evaluation of successful participant blinding concerning their allocation

		Randomization	
		Intervention	Control
Guess	with MI	21	19
	without MI	0	3
	insecure	9	7
